# Tapping into Efficient Learning: An Exploration of the Impact of Sequential Learning on Skill Gains and Learning Curves in Central Venous Catheterization Simulator Training

**DOI:** 10.1177/23821205241271541

**Published:** 2024-10-22

**Authors:** Haroula Tzamaras, Dailen Brown, Jason Moore, Scarlett R. Miller

**Affiliations:** 1 Penn State Department of Industrial Engineering, State College, PA, USA; 2 Penn State Department of Mechanical Engineering, State College, PA, USA

**Keywords:** medical education, simulation, sequential learning, learning curves

## Abstract

**OBJECTIVE:**

Medical residents learn how to perform many complex procedures in a short amount of time. Sequential learning, or learning in stages, is a method applied to complex motor skills to increase skill acquisition and retention but has not been widely applied in simulation-based training (SBT). Central venous catheterization (CVC) training could benefit from the implementation of sequential learning. CVC is typically taught with task trainers such as the dynamic haptic robotic trainer (DHRT). This study aims to determine the impact of sequential learning on skill gains and learning curves in CVC SBT by implementing a sequential learning walkthrough into the DHRT.

**METHODS:**

103 medical residents participated in CVC training in 2021 and 2022. One group (*N* = 44) received training on the original DHRT system while the other group (*N* = 59) received training on the DHRT^sequential^ with interactive videos and assessment activities. All residents were quantitatively assessed on (e.g. first trial success rate, distance to vein center, overall score) the DHRT or DHRT^sequential^ systems.

**RESULTS:**

Residents in the DHRT^sequential^ group exhibited a 3.58 times higher likelihood of successfully completing needle insertion on their first trial than those in the DHRT only group and required significantly fewer trials to reach a pre-defined mastery level of performance. The DHRT^sequential^ group also had fewer significant learning curves compared to the DHRT only group.

**CONCLUSION:**

Implementing sequential learning into the DHRT system significantly benefitted CVC training by increasing the efficiency of initial skill gain, reducing the number of trials needed to complete training, and flattening the slope of the subsequent learning curve.

## Introduction

Central venous catheterizations (CVC) is a commonly performed medical procedure typically performed with ultrasound guidance to insert a catheter into the internal jugular vein (US-IJCVC)^1,2^ for quick medication delivery.^3^ While this procedure is performed more than 5 million times annually in the United States,^4,5^ US-IJCVC is plagued with a high complication rate of 15%,^6^ including complications caused by mechanical errors like accidental puncture of the carotid artery,^7^ or puncturing through the backwall of the vein.^8^ These errors are significant because they can cause complications such as bloodstream infection, stroke, or hemothorax, among others.^3^ The number one driver of these error rates is the experience of the physician performing the procedure—a physician who has performed less than 50 lines is more than twice as likely to incur complications.^6,9^ Therefore, more practice by trainees before transitioning to patients could significantly decrease patient risk, reiterating the need to continuously improve CVC training methods.^9,10^

In order to be successful in US-IJCVC, a sequence of actions requiring both hands and specific motor skills must be followed including: (1) utilizing an ultrasound probe to identify the appropriate vessel, (2) distinguishing the carotid artery from the internal jugular vein on the ultrasound, (3) identifying where to insert the needle with respect to the ultrasound probe, (4) identifying where the needle tip is in the ultrasound image, (5) identifying when the needle has been appropriately centered in the vessel, and (6) understanding how to aspirate the needle and verify when the vein has been accessed.^11^ Current simulation-based training (SBT)^12,13^ in CVC education, while validated in several cases to improve clinical outcomes,^14,15^ typically relies on residents to know and apply these critical steps in order on a manikin without assessing the mastery of skills required for each step.^16^ Skill mastery is an important concept in medical education.^17,18^ The idea of tailoring simulation training to achieving mastery performance has been seen in ventilator management,^19^ bronchoscopy,^20^ and pediatric cannulation,^21^ indicating that training to reach a predefined mastery performance level can improve skill acquisition and knowledge. Additionally, prior research on SBT has indicated that increased feedback may only improve performance in the short term, but not impact longer-term skill retention.^22^

A recent advancement in US-IJCVC training, the dynamic haptic robotic trainer (DHRT), see, relies on comprehensive knowledge assessment. The DHRT system is advanced compared to traditional manikin simulators in that it provides risk-free practice with the ultrasound probe and the needle,^23^ but also allows diverse patient anatomies by changing the locations, depths, and sizes of the IJV and carotid artery.^24^ The DHRT includes a mock ultrasound probe and simulated, reactive ultrasound image, a haptic robot, a specialized retractable needle that provides force to the trainee to simulate insertion, and a feedback screen that provides a personalized performance summary for learning.^25,26^ For each trial on the simulator, the DHRT tracks the aspiration rate, the number of needle insertions, needle centering in the vein, angle of insertion, and puncture through the backwall of the vein or the carotid artery. The system then provides an overall DHRT performance score and a post-trial performance summary screen.^27^ The DHRT has been shown to be as effective as manikin training without the need of a trained preceptor,^28^ and has also been shown to significantly improve learning over time and identify learning curve changes as levels of mastery are reached.^29^ Implementation of the DHRT into the standard curriculum of one large teaching hospital has also been correlated to a decrease in complications at that institution.^30^ However, the DHRT requires residents to apply all facets of their knowledge on their first insertion trial without ensuring an understanding of individual procedural steps. The current training approach for the DHRT is for each resident to conduct 6 preset trials regardless of performance and level of proficiency reached^31^; however, literature indicates that medical education should be tailored to the individual's learning and performance needs^32^ for optimal outcomes.

Assessing the effectiveness of skill mastery is essential in complex motor skills like CVC because prior work has shown that these tasks are best learned in stages rather than all at once.^33^ In fact, there has been extensive research conducted on simulation-based mastery learning in CVC. Mastery-based simulation education in CVC has been demonstrated over the last 15 years by the McGaughy group in the Feinberg School of Medicine at Northwestern University. To do this, the researcher's first sought to define mastery learning for CVC through continuous evaluation and reevaluation of passing standards for clinical skills examinations.^34,35^ After setting validated mastery standards, they applied their simulation-based learning approach and assessed its utility through improvements in quality^36^ and infection^15^ related to CVC. In applying their method, residents were required to meet a minimum passing score and had to continually practice until this score was met.^36^ The utility of the developed method was further proven through the assessment of skill retention, indicating that mastery-based learning for CVC improves skills both in the short- and long-term^37^ and the method was disseminated to other medical centers.^38^ Researcher's continuously monitor the effectiveness of this training method, and have further evaluated the checklist-based mastery education to ensure a reliable, validated measure of clinical competence.^39^

While the McGaghie group succeeded in setting CVC mastery standards for checklist learning and demonstrating that trainees were able to meet mastery performance after rigorous SBT, and in some cases extended practice sessions,^39^ they did not explore multiple types of learning, automation of training, or if implementing different learning methods would allow mastery performance to be reached in less time. Prior work on SBT outside of CVC has shown that medical students prefer learning in a sequential style, or breaking down learning into small steps^40–42^ with an incremental progression of steps.^42,43^ Prior work has also shown that sequential learning is useful for multiple learning styles^44^; however, some literature on sequential learning in SBT is mixed, with one study indicating that the implementation of sequential learning did not significantly impact learning outcomes of medical students for emergency skills after SBT.^45^ Sequential learning has not been explored extensively in SBT for CVC, and research focused on implementing SBT into medical education at the residency level is limited.

Sequential learning has also been shown to increase skill gain and reduce learning curves, or plots that show the number of repetitions required for a trainee to reach a desired level of performance.^46^ The theory of learning curves posits that learning improves with experience, and can be plotted over time.^47^ Learning curves have been studied in SBT. For example, learning curves have been plotted in CVC training^48^ and laparoscopic surgery,^49^ indicating that with SBT learning can improve significantly over a training period. In addition, learning curves have also been shown to differentiate expertise based on performance changes in laparoscopic simulation,^50^ simulated thoracentesis,^51^ and simulated US-IJCVC.^52^ Learning curves also have been found to indicate the efficiency of learning by determining when mastery performance is reached based on curve plateaus, or when the curve flattens^53–56^. Assessing the shape of the learning curve is important to understand the experience of the learner; if the learning curve is flatter, less learning is present whereas if the learning curve is steeper the learner is generally absorbing information and improving their skills.^57,58^ Minimizing and eliminating learning curves of new physicians by pushing them to reach mastery performance faster through robust SBT is important to optimizing patient safety,^58^ as the steepest part of the learning curve would be on the simulator and not on the patient. Applying sequential learning to CVC SBT has the potential to significantly improve learning curves in CVC. Learning curves are also important to explore in the transfer of skills from simulation to living organisms. Previous research comparing learning on a simulator verses on rabbits for laparoscopic surgery found that practice with the rabbits led to higher skill levels when considering live surgery as compared to those who were trained just on the simulated trainer.^59^

To expand the body of knowledge on sequential learning and learning curves in CVC SBT, the purpose of this study was to measure the impact of sequential learning in CVC SBT through assessing initial skill gain, the number of trials and time required for training, and learning curves on the DHRT.

## DHRT^sequential^ Learning Development

The design of the original DHRT system included one 7:30 video that trainees watched at the end of an online training required to be completed prior to attending an in-person training session on the DHRT, and a 27 s refresher video that was played at the start of the in person DHRT training. The 7:30 video outlined how to log into the DHRT, the different parts of the system, and how to use and aspirate the needle. The 27 s refresher briefly re-outlined how to use the DHRT system. Sequential learning was integrated into the DHRT system by breaking down US-IJCVC into 7 key steps developed through guidance by 3 expert physicians and taken from the New England Journal of Medicine (NEJM)^11^ and the National Library of Medicine (NLM).^60^

Specifically, 7 videos were developed as part of the DHRT^sequential^ training ranging in time from 15 to 30 s. In addition, an eighth video was developed to explain the post-trial summary screen and did not include an assessment task. Breaks down the flow of the assessment tasks included in the sequential learning walkthrough training. For each step in the sequential training, a video explanation was provided and followed up with a hands-on activity and assessment that the user had to complete. The initial activities and assessments focused on learning individual skills such as distinguishing the vein from the artery and using the ultrasound probe. These skills were then re-emphasized through learning assessment activities to guide the user through how to conduct all the steps of CVC at once. For example, to teach the skill of using the ultrasound probe, the user would watch a video explaining how to use the probe and then be prompted by the system to use the probe to scan the DHRT^sequential^ surface until they saw the internal jugular vein and carotid artery. The system would not allow them to continue to the next activity until they accomplished this task. Trainees were able to re-watch the explanation video as needed during the assessment activity. The final task and assessment combined all the steps learned throughout the training and had the trainee do an entire practice needle insertion.

In addition to the sequential learning, to better individualize learning to actual performance^32^ the system was also modified for the sequential learning group to only require 3 successful insertions to finish the training depending on the user's score on the performance metrics. The required minimum of 3 trials was found by assessing a subset of previous trainees and finding by which trial residents were beginning to score within the expert range as determined by Pepley in a previous study on the DHRT. This range was identified to be between 80% and 100% with the midrange at 90%.^24^ If the trainee was struggling, they could continue going through trials until they did up to 6 preset trials.^31^ The last trial for each person was referred to as the verification of proficiency (VOP). For the sequential learning group, to finish the training in 3 trials, they had to score anything on the first trial, at least 70% with successful insertion on the second trial, and at least 70% with successful insertion on the VOP. The score required to move forward in training was not set to 100% because of normal variations in the CVC technique that may differ from the optimal score programed into the DHRT. Expert physicians are not likely to score 100% on the DHRT system^24^ due to variations in the angle of insertion, how often the needle is aspirated, and overall procedural flow that depends on the physician, the patient anatomy, and the CVC performance standards within a hospital. Therefore, it would be difficult to expect new trainees to achieve this score. If the 70% requirement was not met, trainees would continue going through the cases until they reached the mastery standard and could continue to the VOP trial, or until they reached trial 5 and were forced to do the VOP for trial 6 regardless of previous score. If participants reached the VOP in 3, 4, or 5 trials but then got less than 70% on the VOP, they would repeat the VOP until they either reached 70% or reached 6 trials.

## Methods

The main objective of this study was to determine the impact of sequential learning in simulator training for CVC based on skill performance, the number of trials, time taken to complete training, and learning curves.

### *Nature of Study*

This study follows a prospective 2 cohort design comparing performance on the DHRT for one group that was exposed to sequential learning, and one group that was not. For the remainder of this paper, the residents who received training on the original DHRT will be referred to as the DHRT only group and those with the sequential learning will be referred to as the DHRT^sequential^ cohort. This study was reviewed by an institutional review board and approved under study number 00012206 in May 2019. This study followed the STROBE checklist^61^ (Supplemental File 1).

Specifically, this study aimed to answer the following research questions (RQs):

**RQ1:** Does the integration of sequential learning in the DHRT impact first-trial success and performance?

Our first research question was developed to examine the impact of sequential learning on resident first-trial insertion performance. Specifically, we sought to understand the impact of the training on resident performance through determining whether the participant was able to obtain venous access without puncturing the carotid artery during their first trial. We hypothesized that training type, distance to vein center, and aspiration rate would be significant predictors of successful performance on the first trial (*H1)* based on the inclusion of sequential learning^42,62^ and prior literature on sequential learning in medical education in laparoscopic SBT.^49^

**RQ2:** Does sequential learning in a CVC simulator impact the number of trials required and performance at the end of training?

The second research question was developed to determine if the inclusion of sequential learning impacted the number of trials conducted, or performance at the end of training, between the DHRT^sequential^ and the DHRT groups. We hypothesized that the number of trials would be significantly different with the DHRT^sequential^ group finishing in fewer trials (*H2*), and that there would be no performance differences for both metrics between groups (*H3)* because both groups should reach mastery performance by their last trial*.* These hypotheses are based on prior literature indicating that complex skills are better learned when broken down^33^ and individualized to the learner,^32^ and that the impact of sequential learning can be seen in mastery level performance being reached faster.^49^

**RQ3:** Does the integration of sequential learning into CVC SBT impact learning curves and time required for training?

The third research question was developed to explore learning curves on the DHRT system between groups for the DHRT performance score based on the number of trials conducted, and to determine if there was a difference in the amount of time required to complete training. We hypothesized that the DHRT^sequential^ group would have significantly fewer learning curves present than the DHRT group (*H4)* and that the overall training time would be reduced *(H5)*. This is because prior research has shown that sequential learning is a better method for efficiently building competence in complex procedures and that competency levels can be differentiated via learning curves.^42,50,56,63^ Additionally, prior research has indicated that the steepness of learning curves can be reduced through incorporating more structure and feedback into learning, and previous studies on the DHRT have indicated that the presence of learning curves on the system signifies learning by the trainee.^29,58^ In the case of sequential learning, the steepest part of the learning curve should be during the initial intervention, thus decreasing the steepness of the learning curve during the trials on the DHRT and creating nonsignificant learning curves.

### *Participants*

To answer our research questions, 103 participants were recruited from residency bootcamps at Hershey Medical Center in 2021 and 2022. Forty-four residents participated in the DHRT group, and 59 residents participated in the DHRT^sequential^ group. A summary of demographic data broken down by DHRT^sequential^ and DHRT groups can be seen in Table 1.

**Table 1.** Demographic summary of residents included in the study.

		DHRT^sequential^	DHRT
Gender			
	Female	25	13
	Male	34	30
	Other	0	1
Specialty			
	Anesthesiology	12	10
	Emergency medicine	9	6
	Internal medicine	14	12
	Other non-surgery	5	7
	General surgery	13	3
	Other surgery	6	6

### *Procedures*

The data collected in this study was part of a larger investigation on residency training and CVC. As such, only the parts of the procedure that are relevant to the current study will be discussed. Before beginning training, residents consented to participate in this study by providing informed written consent prior to training. Next, residents completed an online US-IJCVC training developed through prior work.^64^ Specifically, the training includes 8 video modules with embedded questions focused on teaching: (1) introduction to CVC, (2) an overview of CVC steps as defined by the New England Journal of Medicine,^11^ (3) an overview of the benefits and risks of each access site for CVC, (4) best practices to use CVC equipment, (5) rapid central vein assessment with ultrasound, (6) mechanical procedures for troubleshooting, (7) complication types and how to identify them, and (8) monitoring the patient and removing the catheter.^64^ After completing the online training, participants completed a post-training US-IJCVC knowledge quiz which required a passing score of 80% or higher to attend in person DHRT training. The data from the online training was not used in the current study as it was outside the scope of the current investigation. Once the residents passed this assessment, they watched a 7:30 instructional video on how to use the DHRT system to conduct US-IJCVC. Then, DHRT residents watched a 27 s recap video of how to use the DHRT system and then conducted 6 trials on the system. The DHRT^sequential^ residents underwent the full sequential walkthrough training with activity assessments and then conducted 3 to 6 trials on the DHRT depending on performance. All trials for each group were performed consecutively within a single training session. See the flowchart for a full breakdown of the procedures and how they differed between groups.

## Metrics

The DHRT performance metrics used to answer our research questions included: needle distance to the center of the vein, aspiration rate, successful insertion, DHRT performance score, and last trial performance. These metrics were derived from previous work.^31^ In addition, past training, DHRT performance score, predicted last trial, and patient case were also used. Each of these metrics is defined below.

**Distance to vein center:** This distance is calculated as the radial distance from the tip of the needle at its final position to the center of the vein.^65^ This metric is important because inserting the needle off-center decreases the chance of a successful insertion due to tissue compression and requires more skin incisions to correct. For this variable, a lower number indicates better performance. The ideal score for distance to the center of the vein is 0.

**Aspiration rate:** Aspiration, or pulling back on the plunger of the syringe, is important because the influx of blood into the syringe, referred to as flash, is an indicator to the operator that the vessel has been accessed.^11,24,66^ A higher percentage of aspiration time is beneficial for trainees, so they understand when they have entered the vein; the ideal score for aspiration is 100%.

**Successful Insertion*:*** To have a successful insertion, a participant needed to have ended the trial in the vein *and not* have punctured the carotid artery *or* inserted the needle through the backwall of the vein. An arterial puncture, a backwall puncture, or ending the procedure when the needle is not in the vein resulted in an unsuccessful insertion.

**DHRT Performance Score*:*** For each trial on the DHRT, the participant was given a final score. This score is a combination of their performance metrics and the difficulty of the trial.^27^ The formula to determine the DHRT performance score is DHRT Performance Score = Is* (161.3 * (θs * Cs * as) – Bs – As) where Is refers to if the needle entered the artery or the vein, θs refers to the angle of the needle, Cs refers to the distance to the center of the vein, as refers to aspiration of the needle, Bs refers to puncturing the backwall of the vein, and As is the number of attempts.^65^ If the artery was punctured, the score is automatically changed to zero (failing). A satisfactory score for progressing through the training in fewer trials is 70% or higher with successful insertion.

**Number of Trials:** To complete the training in the DHRT group, each participant was required to conduct between 3 and 6 trials on the DHRT depending on performance. This variable was automatically computed for the DHRT^sequential^ group; however, several participants did not see the GUI indicating they were finished training, so their number of trials was corrected in post-processing based on score. Additionally, to compare the DHRT and DHRT^sequential^ last trial performance, we post-processed the DHRT data by determining which trial the DHRT user would have ended their training based on their performance scores according to the process used on the DHRT^sequential^ group described above.

**Past training:** On the demographic survey conducted in the online training, residents were asked if they have had previous training in CVC. They could answer they had previous training through observation, manikin, robot, other, none, or more than one. Past training was treated as a binary metric for this study.

**Patient Case:** The DHRT system was programed with multiple patient profiles based on hypothetical anatomical variations that could be seen in live patients, as determined by expert physicians.^27^ Each trial conducted on the system was a different patient case, with the first trial and the VOP (last) patient case matching for comparison purposes.

**Training Time**: Training time (in seconds) was measured in the DHRT group as the time to watch the 27 s refresher video and undergo the 6 required trials and in the DHRT ^sequential^ group as the time required to undergo the walkthrough training and the required number of trials based on performance. The time on the system was recorded when a case was actively running and during the walkthrough, and the instructional time was added after training for analysis.

## Results

The main objective of this research was to determine if sequential learning impacts CVC skills gain and learning curves for CVC by answering the following RQs. All statistical analyses for this study were conducted in SPSS (v29).

**RQ1:** Does the integration of sequential learning in the DHRT impact first-trial success and performance?

The first research question was developed to examine the impact of sequential learning on resident first-trial performance through determining if the likelihood of a successful first trial on the DHRT system could be predicted by performance, past experience, and training type of the participant. Our hypothesis was that the inclusion of sequential learning on the DHRT^sequential^ group would significantly improve first-trial success of the sequential learning group.^42^ To test this, a binomial logistic regression was performed with the dichotomous dependent variable, first-trial success, being predicted based on the 2 dichotomous independent variables, past experience and training group (DHRT or DHRT^sequential^), and 2 continuous performance variables, distance to vein center and aspiration rate. Before running the regression model, the percentage of people failing within each group was determined. To achieve a successful insertion, the trainee must have ended the procedure in the vein without puncturing the carotid artery or through the backwall of the vein. In the DHRT group, 28 people (63.6%) failed their first insertion. Of those who failed, 9 (20.4%) failed due to both puncturing the carotid artery and the backwall of the vein, 10 (22.7%) failed due to puncturing through the backwall of the vein, 4 (9.1%) people failed due to ending outside of the vein, and 5 (11.4%) failed due to puncturing the carotid artery. In the DHRT^sequential^ group, 15 (25.5%) people failed their first insertion. Of those who failed, 2 (3.4%) failed due to both puncturing the carotid artery and the backwall of the vein, 9 (15.3%) failed due to puncturing through the backwall of the vein, 1 (1.7%) failed due to ending outside of the vein, and 3 (5.1%) failed due to puncturing the carotid artery.

A post hoc power analysis was conducted in SPSS assuming linear regression and using an effect size of .323 and a predictor value of 4 from the binomial logistic regression in RQ1, resulting in a predicted power of .998. For the binomial regression, prior to computing the analysis, assumptions were verified. Past training was also included as a variable in this analysis to account for different exposure levels of trainees to CVC before attending the DHRT training. The binomial logistic regression model was statistically significant *χ*^2^ (2) = 28.280, *P* < .001 with the model explaining 32.3% (Nagelkerke *R*^2^, a medium effect size^67^), of the variance in successful first insertion and correctly classifying 71.8% of cases. Specifically, the results showed that the training group (Wald *χ*^2 ^= 7.168, *P* = .007) and aspiration (Wald *χ*^2^= 5.177, *P* = .023) were significant predictors of first-trial success while past training (Wald *χ*^2 ^= .047, *P* = .923), and distance to vein center (Wald *χ*^2^= 1.334, *P* = .320) were not. Specifically, DHRT^sequential^ group was associated with an increased likelihood of first-trial success (odds ratio = 3.58, CI 95% [1.41-9.17]) compared to the DHRT group. This result aligns with our hypothesis (*H1)* that DHRT^sequential^ would have a better first-trial insertion performance.

**RQ2:** Does sequential learning in a CVC simulator impact the number of trials required and performance at the end of training?

The primary objective of RQ2 was to determine if the inclusion of sequential learning impacted the number of trials conducted between the DHRT^sequential^ and the DHRT groups or the performance at the end of training. We hypothesized that the number of trials would be significantly different with the DHRT^sequential^ group finishing in fewer trials (*H3*) and that there would be no performance differences for both metrics between groups (*H4).* These hypotheses are based on prior literature indicating that complex skills are better learned when broken down,^33^ and that skill mastery can be achieved through SBT.^21^ provides a cumulative breakdown of the percentages of people finishing the training at each number of trials. For the DHRT^sequential^ group, 64.4% of learners finished in fewer than 6 trials compared to only 38.6% in the DHRT group. To test for differences in the number of trials, a Mann–Whitney U test was conducted. Distributions of performance metrics were similar, as assessed by visual inspection. Results of the Mann-Whitney U test indicated that there was a statistically significant difference in the number of trials performed (*U *= 1012.500, *z* = −2.033, *P* = .042) between the DHRT^sequential^ group (Md = 4) and the DHRT group (Md = 6). A follow-up analysis was conducted on the cumulative distributions of people finishing in each trial group through a Kaplan–Meier survival analysis with a log-rank test. All assumptions were met for this analysis. The results indicated that there was a significant difference (*χ*^2 ^= 5.558, *P* = .018) in the cumulative distributions of people finishing at different timepoints between the people who received sequential learning (*M* = 4.57 trials) and those who did not (*M* = 5.56 trials). Mann–Whitney U tests were also run within each number of trials group for aspiration and distance to vein center to determine performance differences between the DHRT^sequential^ group and the DHRT group. A Bonferroni adjustment was applied to account for testing multiple performance variables,^68^ resulting in an family wise error rate (*α*) of .025. Distributions of performance metrics were similar, as assessed by visual inspection. For distance to vein center in the 3-trial group, the median values were significantly different (*U *= 23.5, *z* = −2.983, *P* = .002) for the DHRT^sequential^ group (Md = .3200) compared to the DHRT group (Md = .4650). No other significant differences were found between groups for either performance metrics. These results align with our hypothesis that the number of trials would be significantly different with the DHRT^sequential^ group finishing in fewer trials (*H2*). These results align with our hypothesis that there would not be significant performance differences at the end of training (*H3*), as a difference was only evident for a single performance metric in the 3-trial group.

**RQ3:** Does the integration of sequential learning into CVC SBT impact learning curves and time required for training?

The objective of this question was to explore learning curves and training time on the DHRT system between groups. Learning was assessed as a group, so logarithmic and linear learning curves were calculated to account for individual differences.^57^ We hypothesized that the DHRT^sequential^ group would have significantly fewer learning curves and less training time than the DHRT group because prior research has shown that sequential learning is a more efficient method for building competence in complex procedures.^42,56,63^ For time, a Mann–Whitney U test was conducted between the total time it took each person to go through the training in each group finding no significant differences (*U* = 1034.0, *z* = −1.760, *P* = .078) between the DHRT group (Md =494.3 s) and the DHRT^sequential^ group (Md = 382.8 s). To study learning curves, curve metric analyses were conducted. For each group, curves were analyzed separately for residents who finished the training in 3, 4, 5, and 6 trials on the system to account for differences in learning speed. Linear and logarithmic curves were fit to each dataset and separately analyzed to determine significance. Specifically, a curve-fit estimation was conducted to identify statistically significant change in performance over time by modeling the DHRT performance score over the number of trials. A Bonferroni adjustment was applied to account for testing for multiple trial groups,^68^ resulting in an family wise error rate (*α*) of .0125. Table 2 shows a breakdown of the results for this section. The DHRT group had 3 significant logarithmic learning curves and 3 significant linear learning curves. The DHRT^sequential^ group had one significant logarithmic learning curve and one significant linear learning curve. See for an example of the linear learning curves graphed for 6-trial groups. The graphs show the mean score for each trial, with the standard deviation as the error bars. The orange line on the graph signifies the expert performance midrange, and the gray line represents the y-intercept of the group. Overall, the learning curves for the DHRT^sequential^ group had flatter slopes compared to the DHRT only group which had steeper slopes. This means that during the trials on the system, more learning was happening for the DHRT only group, whereas the DHRT^sequential^ group did most of their learning during the sequential learning portion of training.

**Table 2.** Curve estimates for each trial in the DHRT and DHRT^sequential^ groups.

	DHRT				DHRT^sequential^			
LOGARITHMIC	*B*	*R^2^*	*F*	*P*	*B*	*R^2^*	*F*	*P*
*3*	48.909	.603	42.598	**<**.001*	9.502	.131	7.054	.011*
*4*	34.854	.426	10.370	.006*	9.108	.095	5.665	.021
*5*	32.794	.385	8.142	.014	13.108	.145	6.466	.015
*6*	14.403	.059	10.000	.002*	2.360	.003	.338	.562
Linear								
*3*	25.250	.521	30.463	**<**.001*	4.875	.118	6.268	.016
*4*	15.725	.399	9.312	.009*	4.893	.126	7.812	.007*
*5*	12.167	.328	6.349	.026	4.950	.128	5.596	.023
*6*	4.564	.073	12.691	**<**.001*	.904	.003	.395	.531

For comparison, shows visual representation of individual linear learning curves for the 2 people ending with the highest scores in the 6-trial groups for the DHRT only and DHRT^sequential^ trainings. Here, each line represents the person's performance trend over the 6 trials. Observing this graph, the DHRT^sequential^ group had higher first trial scores than the DHRT only group. One of the participants for the DHRT^sequential^ group has a downward sloping learning curve; however, this curve was biased by the participant receiving a zero for trial 3 due to puncturing the carotid artery. This person did end the training with a final score of 92%, indicating that they learned and recovered from this mistake. These results align with our hypothesis that there would be fewer significant learning curves in the DHRT^sequential^ group, indicating that trainees started at a higher point and therefore saw less of a significant change from beginning to end of training.

## Discussion

The goal of this study was to measure the impact of sequential learning in simulator training for CVC by assessing the initial skill gain, the number of trials and time required for training, and learning curves on the DHRT^sequential^ compared to on the DHRT alone. The main findings of this study were that

(1) The DHRT^sequential^ group was 3.58 times more likely to have a successful insertion on their first trial, which was significantly impacted by aspiration rate
(2) The number of trials required was significantly different between groups, but performance was not at the end of training
(3) The DHRT^sequential^ group had fewer significant learning curves compared to the DHRT group.

We hypothesized that the implementation of sequential learning would improve CVC simulator training by improving first trial performance (*H1*) on the DHRT, based on previous literature indicating that sequential learning is useful for learning complex skills.^33,49^ Sequential learning did impact the initial success of the procedure by significantly increasing the success rate of learners making them 3.58 times more likely to achieve successful venous access in their first trial. This indicates that the challenge of having to apply multiple skills at the same time on the first use of the system was lessened, and that the exposure and breakdown of skills introduced in the sequential learning walkthrough and assessment activities led to improved initial performance. For specific performance metrics, aspiration rate was a significant predictor of successful first insertion. This was surprising because the DHRT^sequential^ group was explicitly taught how to align the needle to the center of the vein in the assessment activities and how to aspirate throughout the procedure, so it was expected that both skills would have a significant impact on their ability to perform successfully.

We also hypothesized that the number of trials required to finish training would be significantly lower for the DHRT^sequential^ group than for the DHRT only group (*H2*) because of prior literature indicating that complex skills are better learned when broken down^33^ and individualized to the learner.^32^ We also hypothesized that performance would not be significantly different between groups at the end of training (*H3*), because both groups should reach mastery performance by the end of SBT.^21^ The number of trials was significantly different between groups (*P* = .042), as was the difference in the cumulative distributions finishing within each trial number (*P* = .018). The DHRT^sequential^ group had a higher percentage of people finishing in fewer than 6 trials (64.4%) compared to the DHRT group (38.6%), indicating a decrease in trials required to reach a predefined level of mastery with the addition of sequential learning. Additionally, performance was not significantly different between the groups at the end of training, except for distance to vein center in the 3-trial group. Performance not differing at the end of training indicates the utility of individualized learning, and further emphasizes that allowing the required number of trials to range from 3 to 6 depending on performance is a valid training method.

Finally, we hypothesized that the number of significant learning curves and training time would be lessened in the DHRT^sequential^ group (*H4*) due to previous literature indicating the utility of deliberate practice and sequential learning on minimizing procedural learning curves,^49,50,58^ and that sequential learning is a more efficient method for building competence in complex procedures.^42,56,63^ Our findings refute out hypothesis that training time would be less for the DHRT^sequential^ group; however, it is beneficial that there was no difference in training time between groups when considering the extra instructional time added with the walkthrough and the decrease in the number of trials required. Our findings align with our hypothesis that there would be fewer learning curves in the DHRT^sequential^ group. Learning curves were assessed in each training group based on the number of trials and the DHRT performance score. For the DHRT group, there were significant linear and logarithmic curves for all trial groups except for 5 trials. For the DHRT^sequential^ group, the only significant logarithmic curve was for 3 trials, and the only significant linear curve was for 4 trials. This is important because the lack of significant learning curves for the DHRT^sequential^ group coupled with the higher starting performance indicates that the learning curve was steeper during the learning intervention, causing it to flatten out during the actual training trials. More experimentation is needed to validate this. Overall, the learning curves indicated that the DHRT^sequential^ group had less room to improve than the DHRT group, helping to minimize the learning curves for CVC simulation and aligning with prior research on how practice impacts learning in simulation for other procedures.^50,51^ Based on these findings, the addition of sequential learning may allow the learning curve to be steeper during training, and flatter during actual practice on patients leading to a potential increase in patient safety and physician performance.

While these results indicate the potential utility of sequential learning for CVC education and training, this study is just the first step in validating using sequential learning with training to a mastery level as a new learning method. Other approaches to mastery-based learning in CVC, including the gold standard as set by the McGaghie group, started with an in-depth analysis of the standards needed for competency in CVC,^34,35^ and over several studies and years of training continuously validated their method of teaching.^15,36^ To follow this approach, the next steps for this sequential learning method are to measure the long-term skill retention of trainees and to disseminate the method into other medical centers. By following the approach taken by previous researchers which led to a successful mastery-based CVC education with checklists and simulation training, this sequential learning approach with CVC simulation could be a valuable training technique for competency-based learning in CVC.

This study has several limitations that must be addressed. First, this study took place at one US medical center with one specific simulator and as such the generalizability of the findings are limited. Second, the indication that training was completed for the DHRTsequential group on the GUI was not clear to all participants, requiring post-processing to correct. Third, this study assessed learning from a medical simulator for CVC, and as such it is impossible to tell from this study alone if the participants were truly learning the procedure or if they were learning only the device; future work should focus on validating this with a longitudinal study into the clinical environment assessing performance with patients. Expansion to a longitudinal study with more datapoints after training would also allow a better understanding of the forgetting curve and retention of knowledge. The scoring system on the DHRT should also be reassessed to ensure that mistake penalties do not bias the overall picture of learning when assessing the learning curve. Adding to this, this study assessed time differences between cohorts; however, the DHRT cohort spent all their time practicing whereas the DHRT sequential cohort spent part of their time learning with the sequential modules and part of it practicing. As such, future work should explore in more detail if it is the time spent learning or the time spent practicing that makes a bigger impact on learning. Finally, this study focused on the analysis of group learning curves and did not analyze individual learning curves; future work should explore individual learning curves in medical simulation for CVC to determine if findings would differ.

## Conclusion

The goal of this research was to determine if the addition of sequential learning into a CVC simulator could impact learning based on skill performance, the number of trials and time to finish training, and learning curves on the DHRT. The first main finding of this study is that the implementation of sequential learning did significantly impact the initial skill gain through increasing the rate of success of the first trial on a CVC simulator for the DHRT^sequential^ group. Secondly, the number of trials required to complete training was significantly lower for those in the DHRT^sequential^ group. Finally, the DHRT^sequential^ group had fewer significant learning curves. These results demonstrate that the implementation of sequential learning into medical education may be beneficial to improving initial skill gain, decreasing the number of trials required, and minimizing the learning curve during training.

## Supplemental Material

sj-docx-1-mde-10.1177_23821205241271541 - Supplemental material for Tapping into Efficient Learning: An Exploration of the Impact of Sequential Learning on Skill Gains and Learning Curves in Central Venous Catheterization Simulator Training Supplemental material, sj-docx-1-mde-10.1177_23821205241271541 for Tapping into Efficient Learning: An Exploration of the Impact of Sequential Learning on Skill Gains and Learning Curves in Central Venous Catheterization Simulator Training by Haroula Tzamaras, Dailen Brown, Jason Moore and Scarlett R. Miller in Journal of Medical Education and Curricular Development

sj-pdf-2-mde-10.1177_23821205241271541 - Supplemental material for Tapping into Efficient Learning: An Exploration of the Impact of Sequential Learning on Skill Gains and Learning Curves in Central Venous Catheterization Simulator Training Supplemental material, sj-pdf-2-mde-10.1177_23821205241271541 for Tapping into Efficient Learning: An Exploration of the Impact of Sequential Learning on Skill Gains and Learning Curves in Central Venous Catheterization Simulator Training by Haroula Tzamaras, Dailen Brown, Jason Moore and Scarlett R. Miller in Journal of Medical Education and Curricular Development
