# Erratum to “Tapping into Efficient Learning: An Exploration of the Impact of Sequential Learning on Skill Gains and Learning Curves in Central Venous Catheterization Simulator Training”

**DOI:** 10.1177/23821205251346115

**Published:** 2025-05-27

Tzamaras H, Brown D, Moore J, Miller SR. Tapping into efficient learning: an exploration of the impact of sequential learning on skill gains and learning curves in central venous catheterization simulator training. *J Med Educ Curr Dev*. 2024:11. doi: 10.1177/23821205241271541

In this article, figures, in-text citations and their corresponding captions were inadvertently omitted during the production process and are now included in the article.

The missing figures and captions are provided below:

**Figure 1.**
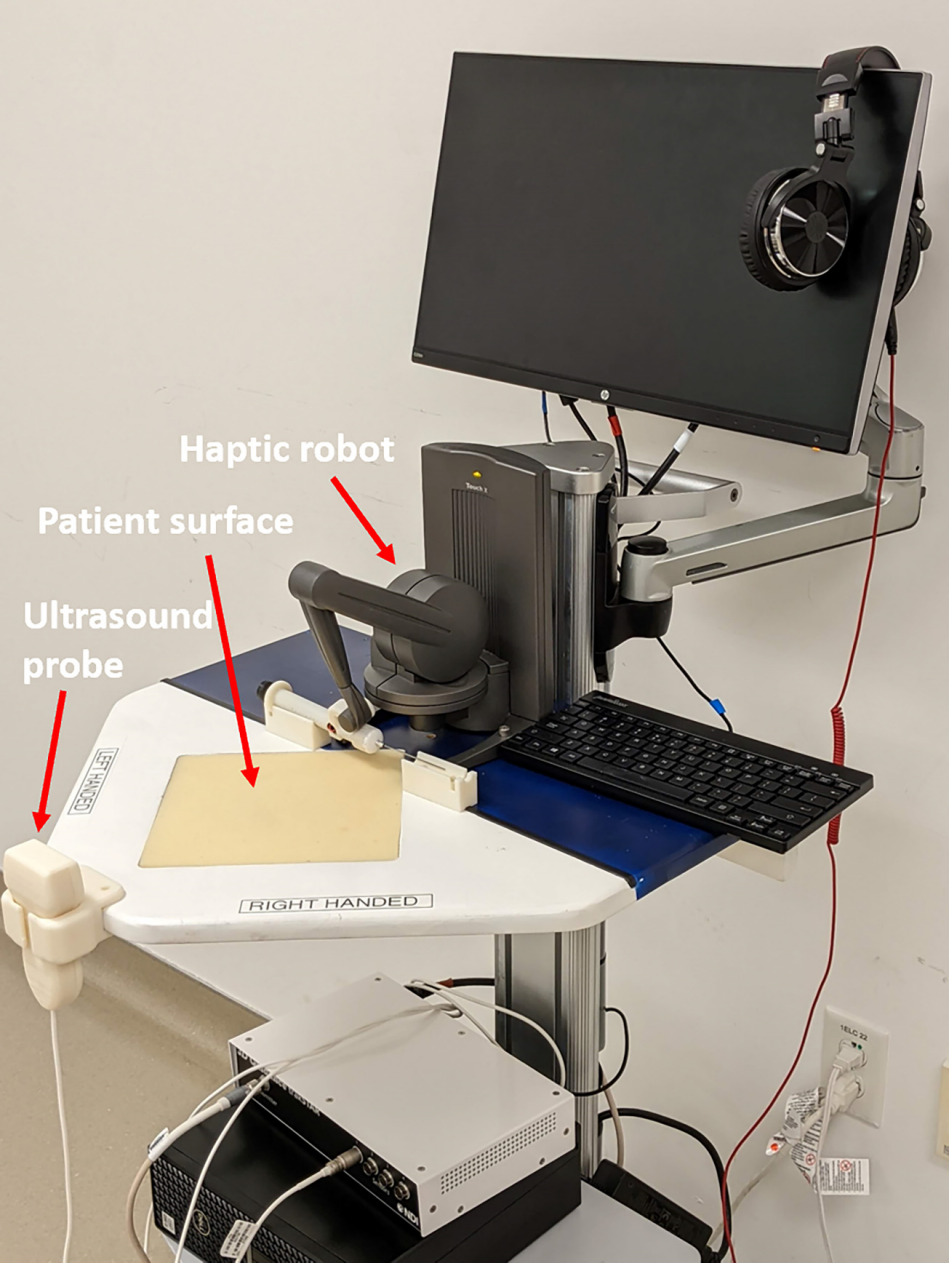
The original DHRT system used for CVC training with parts labelled.

**Figure 2.**
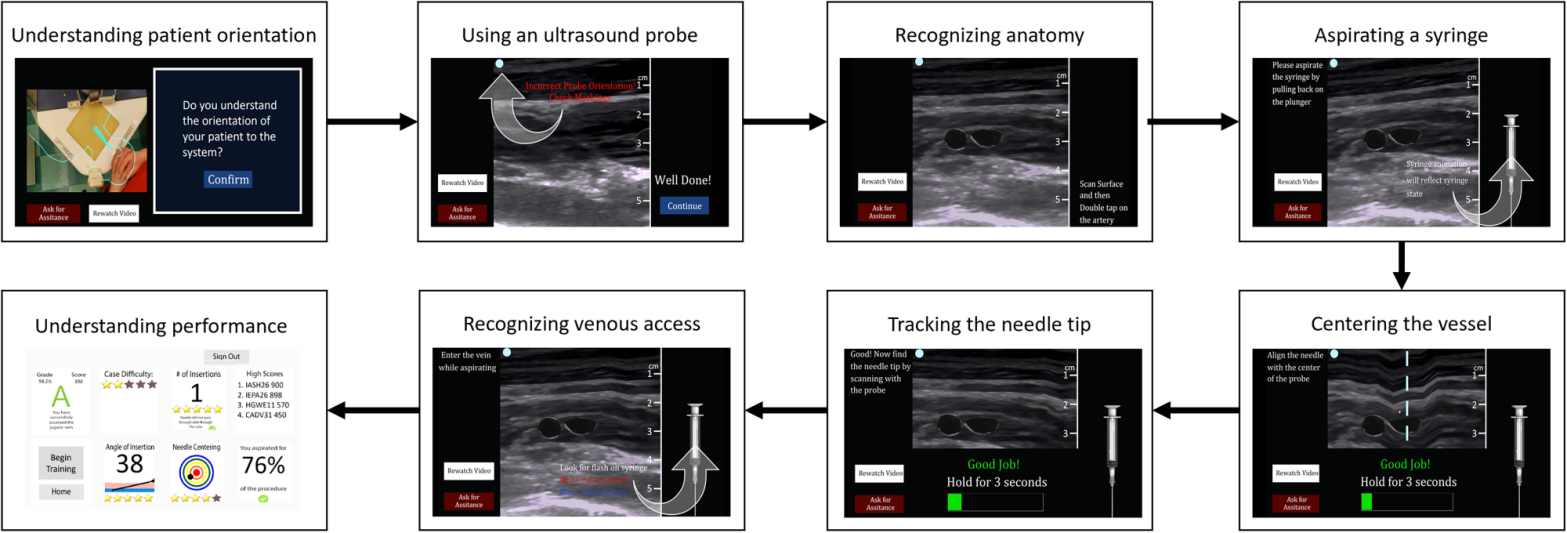
Flow of the learning assessment activities in the sequential learning walkthrough.

**Figure 3.**
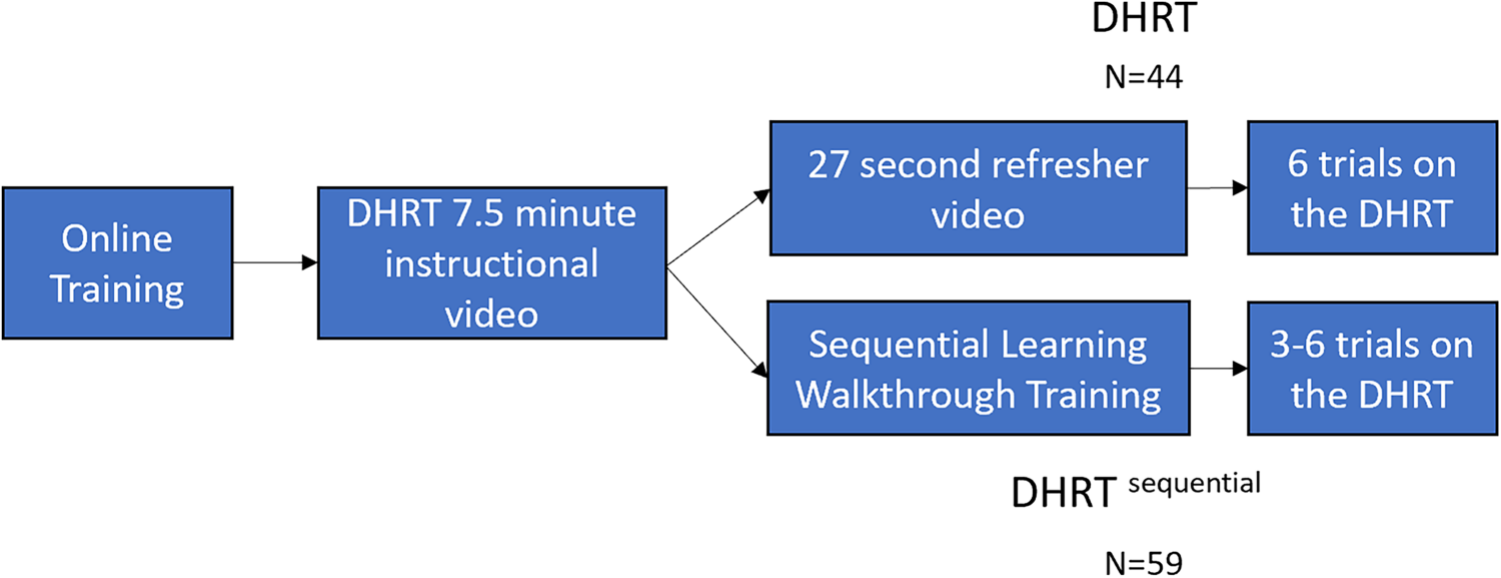
Complete procedural flow for the DHRT and DHRT sequential training groups.

**Figure 4.**
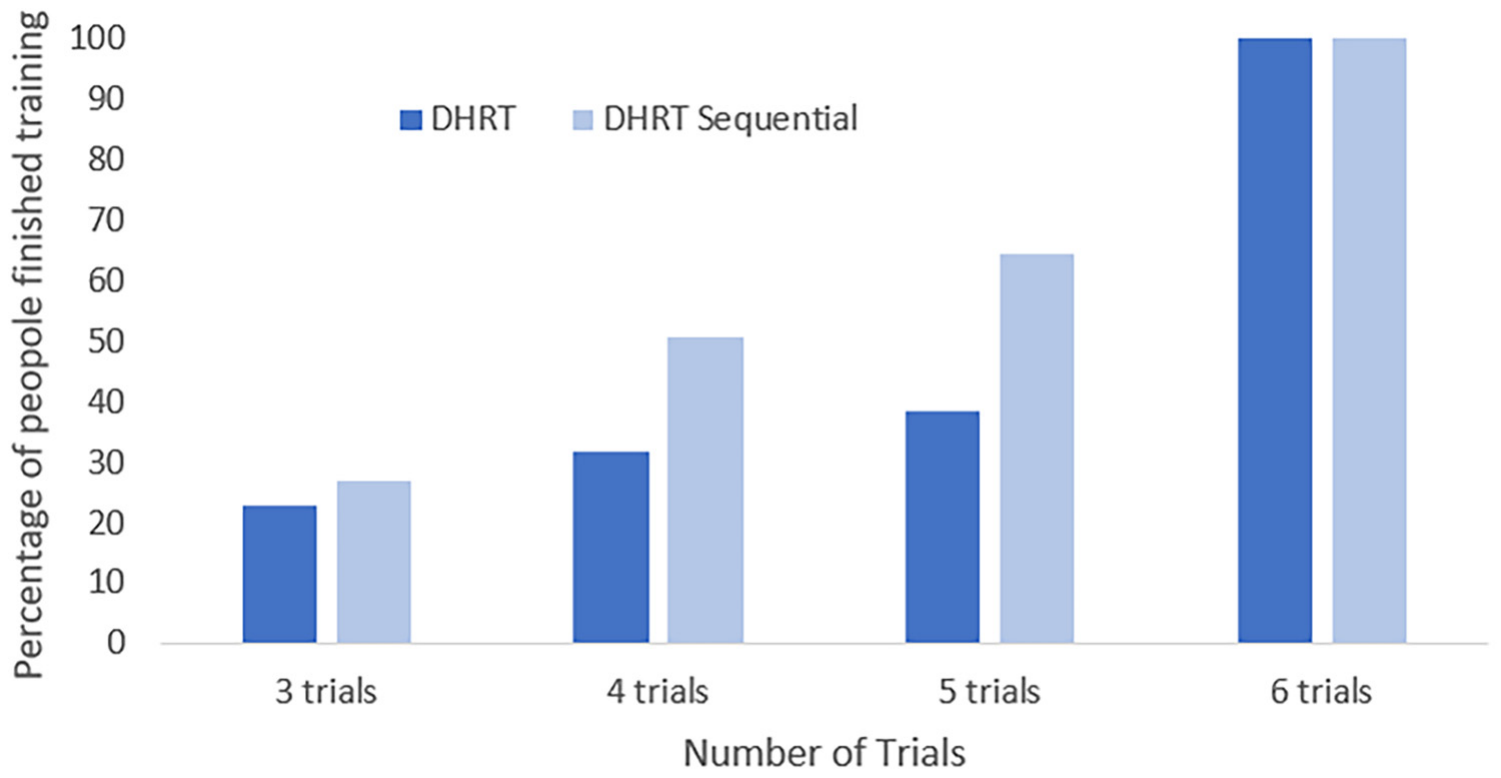
A cumulative percentage graph indicating what percent of each training group was finished at each number of trials.

**Figure 5.**
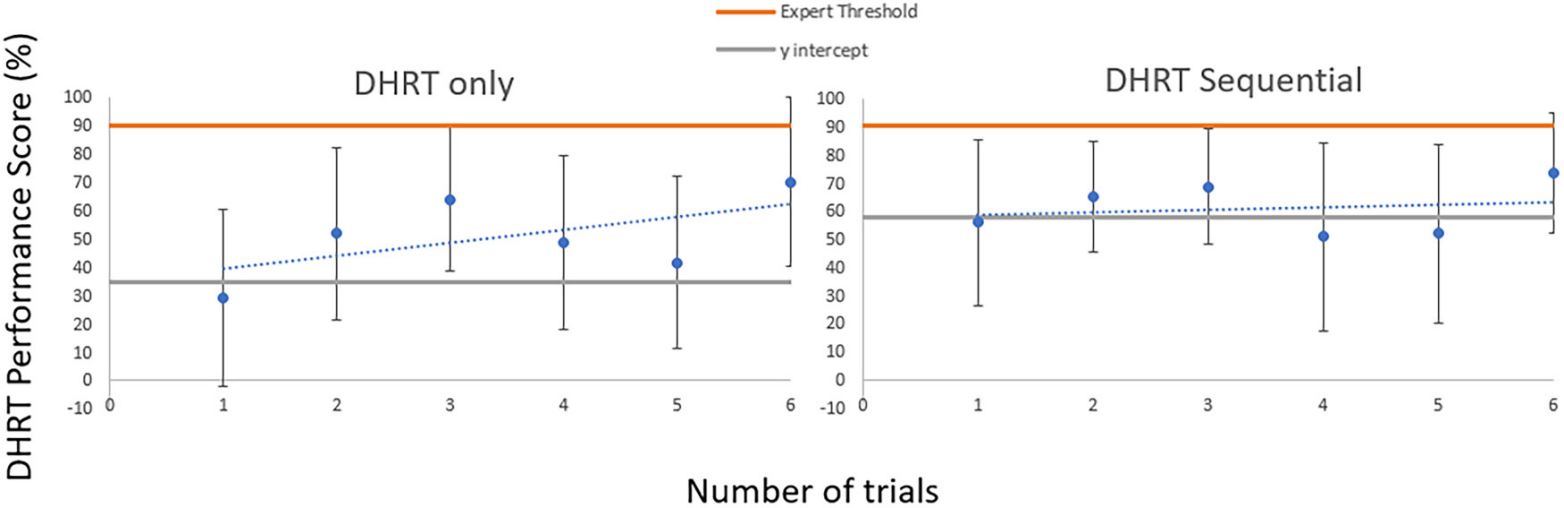
Six-trial group learning curves for the DHRT group (significant) and the DHRT sequential group (nonsignificant).

**Figure 6.**
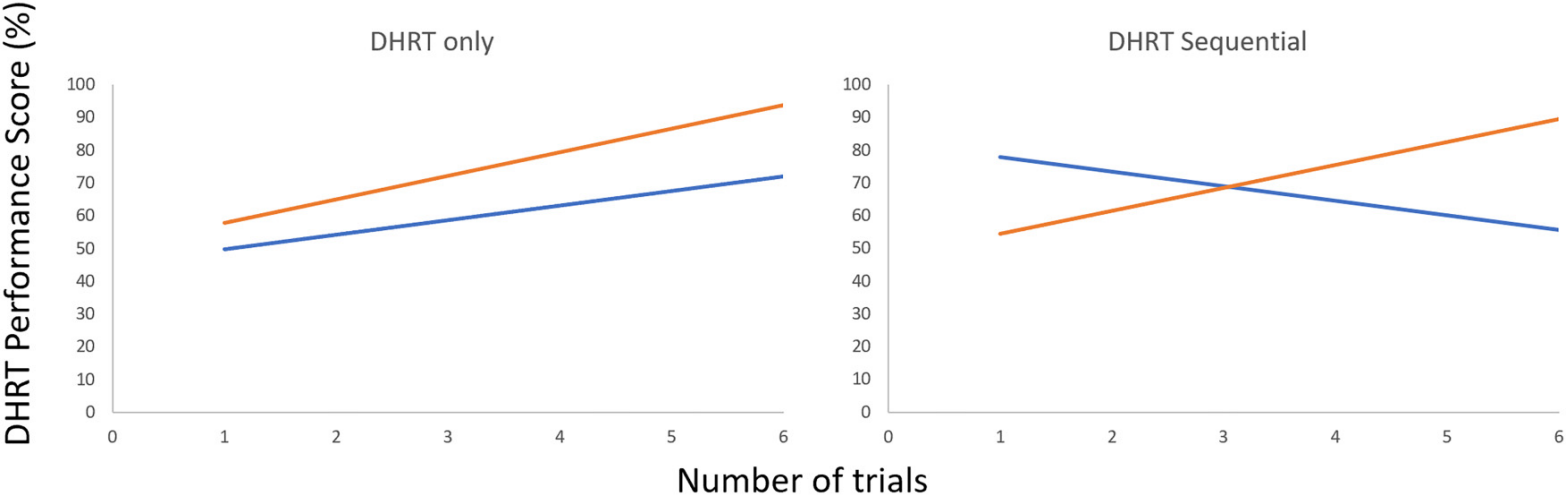
Individual linear learning curves for participants with the two highest scores at the end of training in the six-trial groups.

