# Supplementary material for: Tapping into Efficient Learning: An Exploration of the Impact of Sequential Learning on Skill Gains and Learning Curves in Central Venous Catheterization Simulator Training
Source: J Med Educ Curric Dev. 2024 Oct 22;11:23821205241271541. doi: 10.1177/23821205241271541 (PMC11526281; doi:10.1177/23821205241271541)
Supplement: sj-pdf-2-mde-10.1177_23821205241271541 - Supplemental material for Tapping into Efficient Learning: An Exploration of the Impact of Sequential Learning on Skill Gains and Learning Curves in Central Venous Catheterization Simulator Training [file sj-pdf-2-mde-10.1177_23821205241271541.pdf]

DEMOGRAPHIC QUESTIONNAIRE

April 4, 2019

Area of medical specialty?_______________________________

PGY Level? (Lab residents please count each year in the lab as 1. For example a first year lab resident would be a PGY3) : __________________________

Gender? ☐ Female ☐ Male

Ethnicity?

☐ American Indian/ Alaska Native

☐ Asian

☐ Native Hawaiian or Other Pacific Islander

☐ Black or African American

☐ White

☐ Hispanic______________

☐ More than One Race

☐ Other______________

☐ Prefer not to answer

Please answer the following questions with respect to *__Internal Jugular Catheterization.__*

What type of Central line training have you received (check all that apply):

☐ I have not received any ultra-sound guided CVC training

☐ I received ultrasound guided CVC training on a mannequin (simulator)

☐ I received ultrasound guided CVC training through observations of other procedures (# observed _)

☐ I received CVC training on a haptic robotic simulator

☐ Other training not listed above: ____

How many Internal Jugular Central Lines have you previously completed: #_____

Please answer the following questions with respect to *__any other kinds of catheterization.__*

What type of Central line training have you received (check all that apply):

☐ Subclavian

☐ Femoral

☐ Other training not listed above: ____

How many other kinds of Central Lines have you previously completed?

Type____ # of lines placed_________

Type____ # of lines placed_________

Type____ # of lines placed_________
